# Preoperative imatinib treatment in patients with locally advanced and metastatic/recurrent gastrointestinal stromal tumors: A single-center analysis

**DOI:** 10.1097/MD.0000000000019275

**Published:** 2020-02-28

**Authors:** Jian Wang, Yuan Yin, Chaoyong Shen, Xiaonan Yin, Zhaolun Cai, Lin Pu, Wei Fu, Yaxuan Wang, Bo Zhang

**Affiliations:** Department of Gastrointestinal Surgery, West China Hospital, Sichuan University, Chengdu, Sichuan, China.

**Keywords:** GISTs, preoperative IM therapy, surgical intervention

## Abstract

The advent of imatinib mesylate (IM) has dramatically revolutionized the prognosis of advanced and metastatic/recurrent gastrointestinal stromal tumors (GISTs). The objective of this retrospective study is to investigate the safety and efficacy of combination of surgery following IM treatment in the management of advanced and metastatic/recurrent GISTs. We further explore the long-term clinical outcomes in these who underwent therapy of preoperative IM.

Eligible patients with GISTs before the onset of the IM therapy and were periodically followed up in the outpatient clinic were included in this study. Detailed clinical and pathologic characteristics were obtained from the medical records of our institution. Univariate and multivariate regression analyses were performed to use for the evaluation of potential prognostic factors.

A total of 51 patients were included in the study, of these patients, 36 patients underwent surgery and median duration of preoperative IM is 8.2months (range 3.5–85 months). Significant median tumor shrinkage rate was 29.27% (95% confidence interval 21.00%–34.00%) observed in these patients who responded to IM, and partial response and stable disease were achieved in 24 patients (47.06%) and 23 patients (45.10%), respectively, in light of the RECIST guideline (version 1.1). After the median follow-up of 43.70 months (range 14.2–131.1 months), 1- and 3-year overall survival (OS) were estimated to be 96.1% and 94.0%, respectively, and there was a significant improvement in OS for patients who received surgical intervention versus those who did not.

Our study consolidates that patients were received preoperative IM therapy could shrink the size of tumors and facilitate organ-function preservation. The long-term analysis on this study supports that surgical intervention following IM therapy benefits for patients with primary advanced and recurrent or metastatic GISTs on long-term prognosis.

## 1 Introduction

Gastrointestinal stromal tumor (GIST) is the most common mesenchymal tumor of the gastrointestinal tract which arises from the interstitial cells of Cajal.^[1,2]^ The introduction of imatinib mesylate (IM), a receptor tyrosine kinase inhibitors (TKIs) of KIT, and platelet-derived growth factor receptor-alpha (PDGFRA), has revolutionized the management of GISTs.^[3]^ Although complete surgical intervention followed by IM therapy has become the primary method in management of patients with Intermediate, high-risk GISTs, while the site and/or size of the GISTs can lead to surgical resection difficult, requiring complex operations or even posing permanent lifestyle changes.^[4]^ A series of studies have demonstrated that preoperative IM treatment could effectively shrink tumor and reduce surgical morbidity in patients with primary unresectable or resectable GISTs through a major surgical procedure with significant surgical morbidity.^[5–17]^ However, the long-term clinical outcomes of preoperative IM administered for patients with recurrent and/or metastatic GISTs remain uncommon. The objective of this study was to display our single-center experience on preoperative IM therapy for the patients GISTs to guide the management of these complex GIST patients.

## 2 Materials and methods

### 2.1 Patient selection and management

Eligible patients with histologically proven GISTs at our institute were enrolled in the study, from January 2008 to April 2016. The inclusion criteria are the following: Patients with primary advanced GIST lesions, the site and/or size of the GISTs can make surgical resection difficultly, requiring perplexing surgical intervention, or leading to permanent lifestyle changes. For the aim of this analysis, these patients will be classified as group A. The patients with recurrent and/or metastatic GISTs, identified as the presence of tumor recurrence demonstrated by histology or radiography after last surgery of the GIST, before the time of the initiation of IM preoperative treatment, the patients did not undergo any other GIST-specific drugs treatment and these patients will be defined as group B.

The patients were followed up periodically in our specialized outpatient of GIST during IM medication. The medical history was obtained along with performance with clinical examination and CT (computed tomography)/MRI (magnetic resonance imaging) scan. Meanwhile clinical data such as demographic data, status of disease, clinical presentation, response to treatment, surgical condition, mutation type, and postoperative complications were also collected. The retrospective analysis of data involved in this study is anonymous and has been approved by the West China Hospital Research Ethics Committee.

### 2.2 Preoperative treatment, surgical intervention

Management with standard-dose IM (400 mg daily) in initial dose was the first choice of treatment in all enrolled patients. The objective response (tumor shrinkage) assessment of IM therapy is in light of the Response Evaluation Criteria in Solid Tumor (RECIST 1.1) and the optimum IM treatment response was defined as a complete response partial response (PR), stable disease (SD). Since the optimal duration (or plateau response) of preoperative IM therapy remains unknown, in patients with diseases who responded to IM therapy, IM should be continued until best response (defined as no further change between 2 successive CT and/or MRI scans). When the best response arrived, surgical intervention would be taken into consideration. In our institution; however, the final decision for surgery would carefully be made by multidisciplinary team, which consisting of radiologists, gastrointestinal surgeons, oncologists and pathologists. All surgical resection was classified as R0 (complete removal of tumor tissue with negative microscopic margins), R1 (removal of tumor tissue with positive microscopic margins) or R2 (macroscopically incomplete resection).

### 2.3 Statistical analysis

Overall survival (OS) was defined as from IM introduction to death or last follow-up occurred. Progression-free survival (PFS) was defined as from start of IM to death or relapse or last follow-up, whichever occurred first. PFS and OS estimates and standard errors were obtained by the Kaplan–Meier method and the log-rank test was used to compare differences between the curves. In addition, univariate and multivariate analyses were performed with stepwise Cox proportional hazards regression modeling for identification of clinical prognostic factors for PFS and OS. Test-statistical comparisons were performed using Chi-square test, Fisher exact, or Willxon rank-sum test as appropriate for comparisons. A 2-sided *P* value <.05 was considered statistically significant. All data analysis was performed using the program GraphPad Prism 8.02 (San Diego, CA) for Windows.

## 3 Results

### 3.1 Patients characteristics

The baseline characteristics of these patients are depicted in Table 1. There were 31 patients (60.8%) in group A (primary advanced GIST) and 20 patients (39.2%) in group B (metastatic and/or recurrent GIST). The majority of primary GIST presented in the stomach (35.5%), followed by rectum (29.0%), small bowel (16.1%) in group A; for patients in group B, 12(60%) patients with recurrent and/or metastatic lesions after previous surgery, 8 (40%) patients at the time of initial diagnosis concurrent liver and/or peritoneal metastasis. Regarding the mutational analysis, 33 (65%) patients were available, 25 patients (49%) had KIT exon 11 mutations, 5 patients (10%) were KIT exon 9 mutations, 3 patients (6%) were wild type (WT).

**Table 1:**
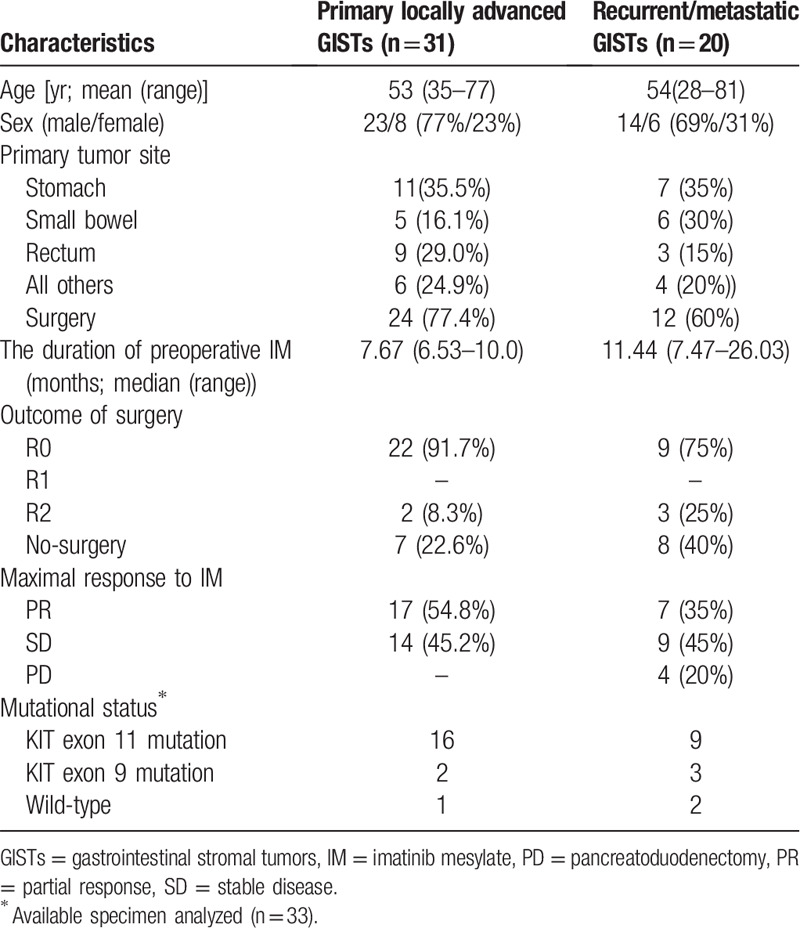
The baseline characteristics of patients (n = 51).

### 3.2 Preoperative treatments evaluation

**Figure 1:**
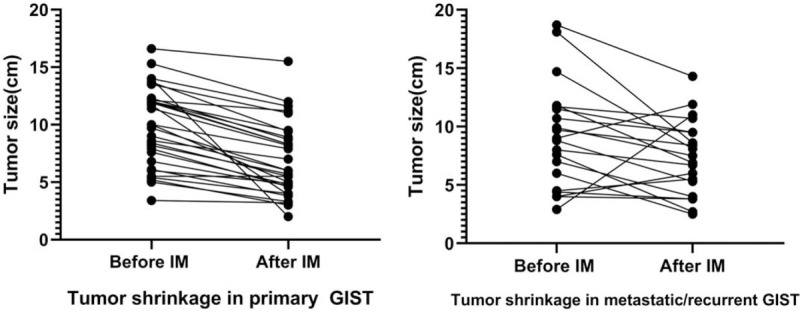
Tumor shrinkage after preoperative in locally advanced and metastatic/recurrent GISTs. GISTs = gastrointestinal stromal tumors.

**Figure 2:**
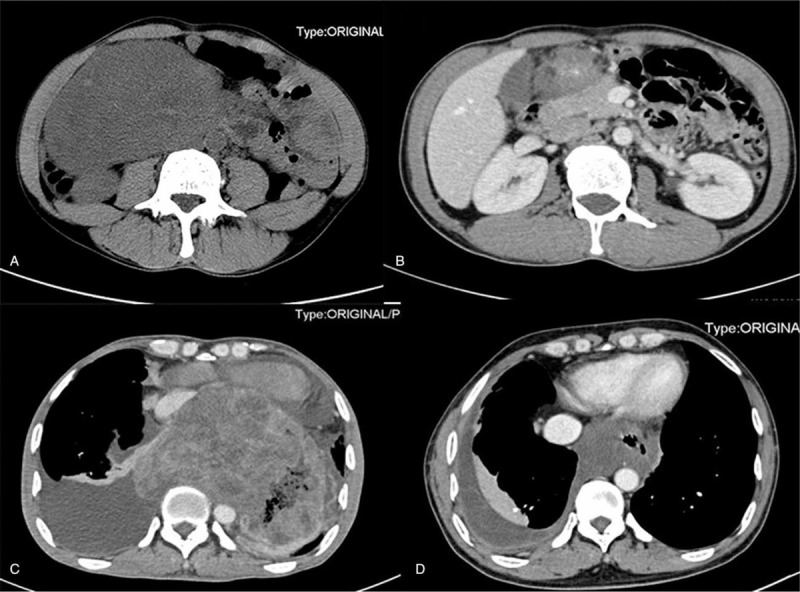
Tumor shrinkage at radiological appearance. (A, B,) A 14-cm lesion at the duodenal second portion (A) reduced to 2.7 cm (B) after months preoperative IM therapy. A 11.7 cm-tumour (C) in esophago-gastric junction had shrunk to the size of a 3.9 cm (D) through duration of 10-mo preoperative IM. IM = imatinib mesylate.

According to the RECIST criteria (1.1), 17 patients (54.8%) had a PR, 14 patients (45.2%) had SD as their best response in group A, while patients had PR, SD and PD were arrived in 7 patients (35%), 9 patients (45%), 4 patients (20%), respectively, in group B. The significant median tumor shrinkage rate was 29.27% (95% confidence interval [CI] 21.00%–34.00%) observed in these patients (n = 47) who responded to IM and the median diameter of tumor reduced from 9.9 cm to 5 cm (95% CI 8.2–11.7 cm; 95% CI 5.0–8.3 cm, *P* < .0001) (Figs. 1 and 2).

### 3.3 Surgery and postoperative treatment

Among all patients, 36 (70.6%) patients underwent surgery. The detailed information of surgical procedures was summarized in Table 3. Surgery-related complications were observed in 5 (13.9%) patients and included anastomotic fistulas (n = 2), postoperative ileus (n = 2), wound dehiscence (n = 1). It is important to note that patients in group B tend to have higher risk of postoperative complications and longer postoperative hospital stay compared with that in group A (4/ 12 vs 1/24, *P* = .036; 19.02 days vs 9.83 days, *P* < .0001). There were no perioperative deaths happened. Six patients with continuation of IM treatment finally switched to sunitinib therapy because of disease progressing or postsurgical recurrence (Table 2).

**Table 2:**
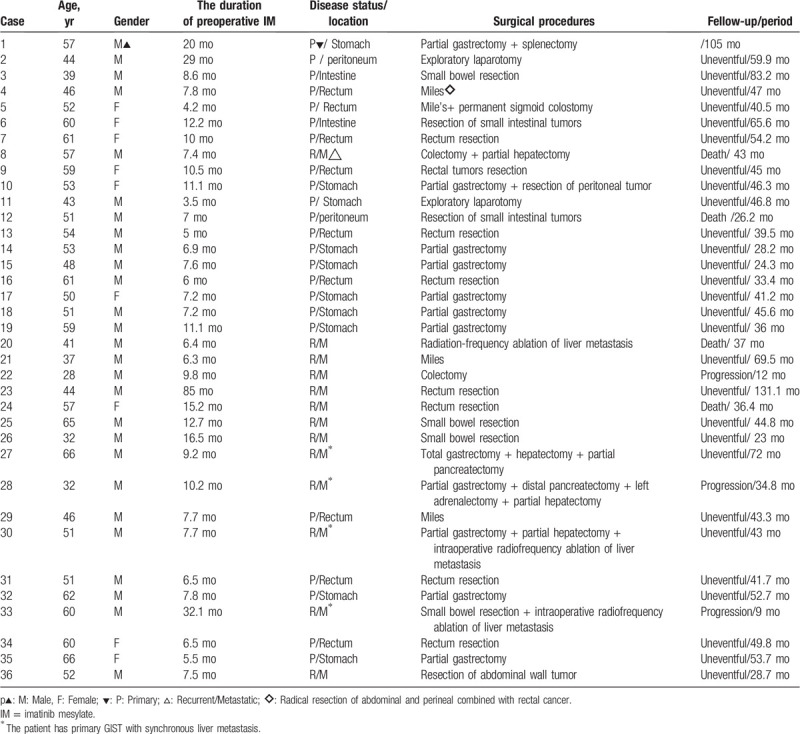
The content of surgery.

### 3.4 PFS and OS in patient subgroups

**Figure 3:**
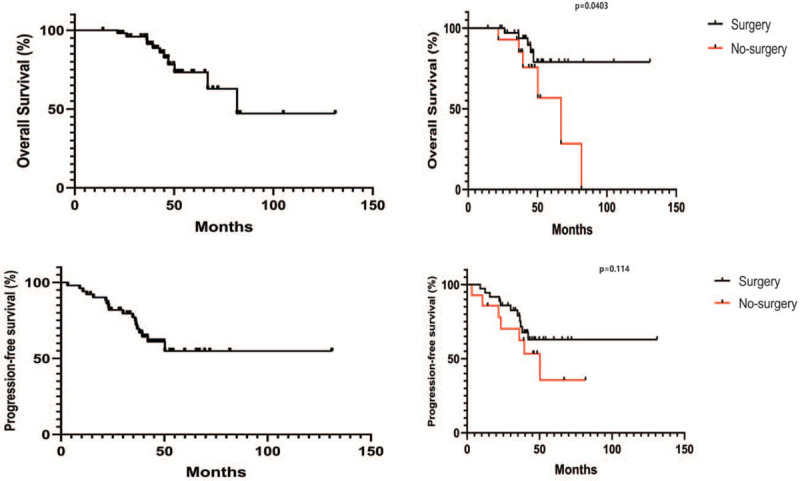
Kaplan–Meier estimates of overall survival (OS) and progression-free survival (PFS) for eligible patients in preoperative IM. IM = imatinib mesylate.

After the median follow-up of 43.70 months (range 14.2–131.1 months). The median PFS in these patients (n = 47) who responded on IM was not reached while 1-, 2-, and 3-year OS was estimated to be 95.7%, 80.3%, and 72.7%, respectively. The median OS from the time of onset of IM was estimated to be 69.5 months (not reached) (Fig. 3). By univariate analyses and multivariable analyses, surgical intervention seemed to play a pivotal role in advanced GIST (Table 3).

**Table 3:**
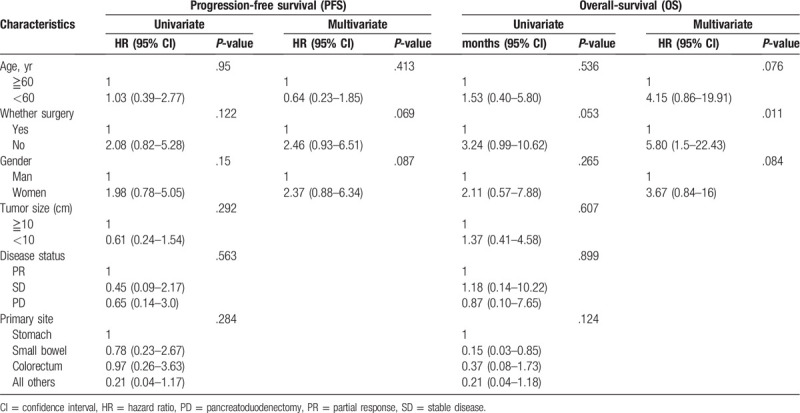
Univariate and multivariate analyses of prognostic factors for progression-free survival (PFS) and overall survival (OS).

## 4 Discussion

The prognosis of these patients with locally advanced and metastatic/recurrent GIST has been dramatically improved by IM and it has been widely accepted as first-line systemic therapeutic strategy.^[1]^ The RTOG 0132/ACRIN 6665 is the first prospective study to demonstrate the feasibility of preoperative IM.^[17,18]^ The long-term oncological outcome of preoperative IM for locally advanced diseases has been reported. In a retrospective study, Tielen and colleagues reviewed 57 patients and found that combining IM and surgical intervention in patients with locally advanced GIST seemed to improve PFS and OS compared with available historical reported series.^[11]^ Additionally, Mussi et al analyzed 80 patients and found that metastasectomy may benefit for the patients with GIST response to IM on survival compared with patients treated with IM alone in historical published reports.^[5]^

Cytoreduction with IM may facilitate the rate of R0 resection and function-sparing surgery. In case of rectal GISTs, preoperative therapy made sphincter-preserving surgery to be undertaken. The efficacy for quality of life is appealing if preoperative IM could preserve the anal sphincter and avoid permanent lifestyle changes (such as permanent colostomy), but it ought to be borne in mind that the clinical situations such as tumor localization or other factors can make it difficult.^[13,19]^ In this study, of 10 patients with rectal GISTs who received surgical resection, 1 underwent permanent sigmoid colostomy for the purpose of R0 resection. Similarly, duodenal GIST should be approached via excision if procedure of pancreatoduodenectomy would be required to achieve a negative histologically margin resection, then preoperative IM should take into consideration.^[20]^

With regard to metastatic and/or recurrent GIST, in the pre-IM era, surgery for patients with metastatic and/or recurrent GIST was not associated with a favorable outcome, which has been significantly changed with introduction of adjuvant IM treatment.^[5,21,22]^ However, it is apparent that most patients who initially response to IM treatment eventually acquire secondary progression, and median time from disease control to progression is approximately less than 2 years reported by some previous large clinical trials.^[23]^ The purpose of surgical resection of recurrent and/or metastatic lesions that response to IM is to prevent potential development of secondary mutations which is believed the main cause of progression. Furthermore, surgical removal of IM-resistant or unresponsive GISTs may contribute to prolonging the duration of disease control. Consistent with previous trials, the patients underwent surgical removal of the metastatic lesion may improve the outcome of advanced GIST patients compared to IM treatment alone. Besides, elimination of resistant lesion is believed in favor of reintroduction of IM management in the context that second-line therapies are frequently not as well-tolerated as that of IM.^[24–26]^ Concerning synchronous/metachronous liver metastases, Y-Jiang Ye and colleagues found that combination of surgery with TKI treatment may be the most effective strategy for GIST patients with liver metastases.^[27]^ It has been reported in a retrospective study shown that surgical resection of liver metastases and primary lesion in GIST patients combined with IM may be associated with prolonged OS.^[28]^ However, in our study, the short-time outcomes of surgical intervention in metastatic and/or recurrent GIST suggested that surgical intervention for these patients is difficult. Therefore, careful consideration of surgical options in patients with liver metastases should be determined on a patient-to-patient base in case of postsurgical complications.

Since the optimal duration of preoperative therapy remains unknown. In this study, median duration of preoperative IM is 8.2months (range 3.5–85 months) which is in-line with the previous published reports.^[16,17,29]^ The National Comprehensive Cancer Network guidelines and Asian Consensus Guidelines have recommended patients with disease that is responding to IM, should continue IM until reaching best response to IM which defined as no further improvement between 2 successive via CT/MRI scans.^[30,31]^ Concerning mutation analysis, there is an increasing number of studies support that testing for mutations in KIT and PDGFRA when determine treatment strategy especially before beginning preoperative IM to ensure tumor has a mutation type that is likely to respond to IM. Generally, KIT exon 9 mutations can benefit from higher dose of IM or second-line treatment, sunitinib, while PDGFRA D842 V mutations or WT mutations that lack of mutation in KIT or PDGFRA cannot benefit from IM therapy compared to KIT exon 11 mutations.^[32]^ Recently, avapritinib (also called Blu-285), a highly selective and potent a type I KIT/PDGFRα inhibitor, has shown great safety and efficacy in management of GISTs with PDGFRA D842 V mutations.^[33]^ In addition, Cai et al, first reported that a patient with sunitinib-resistant GIST regained disease control after introduction of apatinib, a novel, small molecule, selective vascular endothelial growth factor receptor-2 TKI.^[34]^ Unfortunately, in the present study, we did not analyze the relationship between mutational status and efficacy of preoperative IM therapy. The possible reasons may be that the mutation information of patients in this study was limited by their economic status and wills.^[28–35]^

There were several shortcomings do exist in this study. As a retrospective study, selection bias is unavoidable. Therapeutic strategy in patients with good performance status was prone to undergo surgical intervention instead of IM treatment alone.

## 5 Conclusions

In summary, the long-term outcome of our study shown that preoperative IM followed by surgical intervention may benefit for patients with primary advanced and recurrent and/or metastatic GISTs even local progression.

## Acknowledgments

The authors gratefully acknowledge the whole staff of the Department of Gastrointestinal Surgery, West China Hospital, who generously provided assistance in the collection of data throughout the duration of the study.

## Author contributions

**Conceptualization:** Yuan Yin, Bo Zhang.

**Data curation:** Jian Wang, Lin Pu, Yaxuan Wang, Wei Fu.

**Methodology:** Yuan Yin, Zhaolun Cai, Chaoyong Shen,Xiaonan Yin.

**Supervision:** Bo Zhang.

**Validation:** Bo Zhang.

**Writing – original draft:** Jian Wang, Yuan Yin.

Bo Zhang orcid: 0000-0002-0254-5843.
